# BATF regulates the development and function of IL-17 producing iNKT cells

**DOI:** 10.1186/1471-2172-14-16

**Published:** 2013-03-27

**Authors:** Kimberly L Jordan-Williams, Stacie Poston, Elizabeth J Taparowsky

**Affiliations:** 1 Department of Biological Sciences and Purdue University Center for Cancer Research, Purdue University, West Lafayette, IN 47907, USA; 2 Department of Biological Sciences, Hansen Life Sciences Research Building, Room 219, 201 South University Street, West Lafayette, IN, USA

**Keywords:** BATF, Activator-protein-1, iNKT cells, IL-17, Mouse models

## Abstract

**Background:**

BATF plays important roles in the function of the immune system. *Batf* null mice are deficient in both CD4^+^ Th17 cells and T follicular helper cells and possess an intrinsic B cell defect that leads to the complete absence of class switched Ig. In this study, Tg mice overexpressing BATF in T cells were used together with *Batf* null mice to investigate how altering levels of BATF expression in T cells impacts the development and function of a recently characterized population of iNKT cells expressing IL-17 (iNKT-17).

**Results:**

BATF has a direct impact on IL-17 expression by iNKT cells. However, in contrast to the Th17 lineage where BATF activates IL-17 expression and leads to the expansion of the lineage, BATF overexpression restricts overall iNKT cell numbers while skewing the compartment *in vivo* and *in vitro* toward an iNKT-17 phenotype.

**Conclusions:**

This work is the first to demonstrate that BATF joins RORγt as the molecular signature for all IL-17 producing cells *in vivo* and identifies BATF as a component of the nuclear protein network that could be targeted to regulate IL-17-mediated disease. Interestingly, these studies also reveal that while the *Il17a* gene is a common target for BATF regulation in Th17 and iNKT-17 cells, this regulation is accompanied by opposite effects on the growth and expansion of these two cell lineages.

## Background

BATF is a basic leucine zipper transcription factor that dimerizes with the JUN proteins to direct patterns of activator protein-1 (AP-1)-mediated gene expression in the immune system [1]. The impact of disrupting BATF function *in vivo* has been examined by several groups [2-5]. Mice in which BATF is overexpressed using a T cell-specific promoter display a reduced number of iNKT cells [6], an increased number of CD4^+^ T cells expressing IL-17 (Th17) [5] and an altered cytokine environment that promotes the gross overproduction of class switched Ig by B cells [7]. *Batf* null mice are viable, yet display a severe deficiency in Th17 and T follicular helper cells [2,3,5]. The T cell deficiencies are combined with an intrinsic B cell defect blocking the production of class switched Ig to impair the immune response of these animals to antigen challenge [2,3]. The dramatic consequences of altering BATF expression *in vivo* provides evidence that BATF functions to coordinate immune system activities critical in autoimmunity, inflammation and the host response to pathogens.

The ability of BATF to promote the differentiation of naïve CD4^+^ T cells to the Th17 lineage has been shown to rely on the formation of IRF4/BATF protein complexes that bind and transactivate a number of genes, including *Il17a/f*[8]. Interestingly, we have observed a negative influence of BATF on the development of iNKT cells [6,9] and therefore sought to examine how these two opposing activities of BATF may influence the development of a recently identified subset of iNKT cells that expresses IL-17 [10-13]. Murine iNKT-17 cells are a CD4^-^ NK1.1^-^ population that is enriched in peripheral LN (PLN) and respond following stimulation by rapidly secreting IL-17. iNKT-17 cells express RORγt and additional markers that define them as a lineage distinct from classic iNKT cells. A role for iNKT-17 cells has been demonstrated in experimental models of airway disease, asthma and collagen-induced arthritis [12,14,15]. iNKT-17 cells are over-represented in NOD mice where their influence in the pancreas exacerbates the development of diabetes [16]. In the present study, using mouse models of BATF overexpression (*CD2-HA-BATF*) and deficiency (*Batf*^*ΔZ/ΔZ*^), we demonstrate the importance of BATF to the development of iNKT-17 cells. Despite the overall reduction in the number of iNKT cells in *CD2-HA-BATF* mice, the majority express IL-17. Likewise, while peripheral iNKT cell numbers are increased in *Batf*^*ΔZ/ΔZ*^ mice, the cells are deficient in IL-17 production. These data are consistent with results in Th17 cells [5,17] and suggest that BATF-containing protein complexes transactivate the *Il17a/f* gene in NKT cells as well. The novel finding is that the function of BATF as an IL-17 inducer is separate from its effect on cell growth since Th17 cell numbers are expanded in the presence of BATF, while iNKT cell numbers are reduced. To identify an *in vitro* system that would facilitate the study of BATF-mediated gene regulatory events relevant to the iNKT cell lineage, we describe features of the DN32.D3 hybridoma [9] that indicate similarity to the iNKT-17 lineage, including the BATF-dependent expression of *Il17a* mRNA. We conclude that BATF joins RORγt as the molecular signature for all IL-17 producing cells *in vivo* and represents an essential component of a nuclear protein network that could be targeted to regulate IL-17-mediated disease.

## Results and discussion

### BATF controls CD4^+^ Th17 cell differentiation

**Figure 1:**
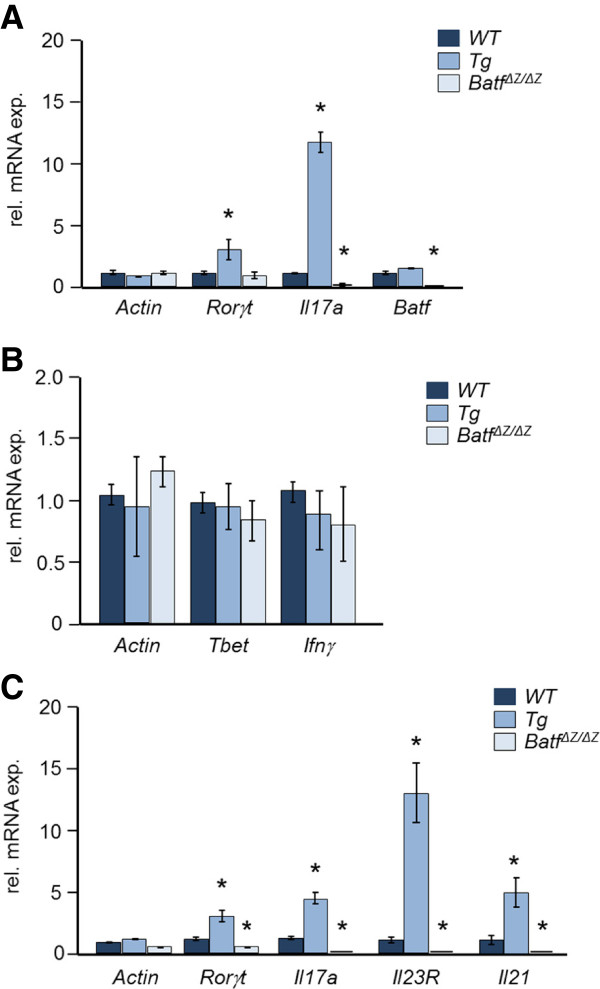
**BATF impacts Th17 differentiation *in vivo *and *in vitro*. A** RNA prepared from stimulated, CD4^+^ T cells of the indicated genotypes was analyzed for Th17-associated transcripts by qRT-PCR. **B** and **C** Naïve CD4^+^ T cells of the indicated genotypes were cultured *in vitro* under conditions skewing differentiation to Th1 (**B**) or Th17 (**C**) cells. After 5 days, RNA was analyzed for the indicated transcripts by qRT-PCR. Data were averaged from 6 (n = 6) (**A**) or 3 (n = 3) (**B** and **C**) mice per genotype. Bars indicate standard error. *p values < 0.05.

To test if *CD2-HA-BATF* Tg mice display the increase in IL-17 expression reported for a separate model of BATF overexpression *in vivo *[5], splenocytes were stimulated with anti-CD3 and anti-CD28 antibodies and RNA isolated after 48 h. For comparison, cells from non-Tg (WT) and *Batf*^*ΔZ/ΔZ *^mice were used. Gene expression analysis by quantitative (q)RT-PCR revealed the expected high level of *Il17a* mRNA in Tg cells and undetectable levels of *Il17a* mRNA in *Batf*^*ΔZ/ΔZ*^ cells (Figure 1A). To implicate altered Th17 development as the major contributor to this change in *Il17a* expression, naïve CD4^+^ splenocytes from Tg, WT and *Batf*^*ΔZ/ΔZ *^mice were subjected to *in vitro* protocols skewing differentiation toward the Th17 lineage or toward Th1 cells as a control. Differentiation was assessed by measuring the expression of genes specific for these CD4^+^ T cell subsets. As shown in Figure 1B, levels of Th1 associated transcripts (*Tbet* and *Ifnγ)* were statistically similar across all samples, while levels of Th17 associated transcripts (*Rorγt, Il17a, Il23R* and *Il21)* were elevated in Tg samples and essentially undetectable in *Batf*^*ΔZ/ΔZ *^samples (Figure 1C). These findings in *CD2-HA-BATF* mice confirm a positive role for BATF in the differentiation of Th17 cells.

### Levels of BATF correlate with altered iNKT cell numbers *in vivo*

**Figure 2:**
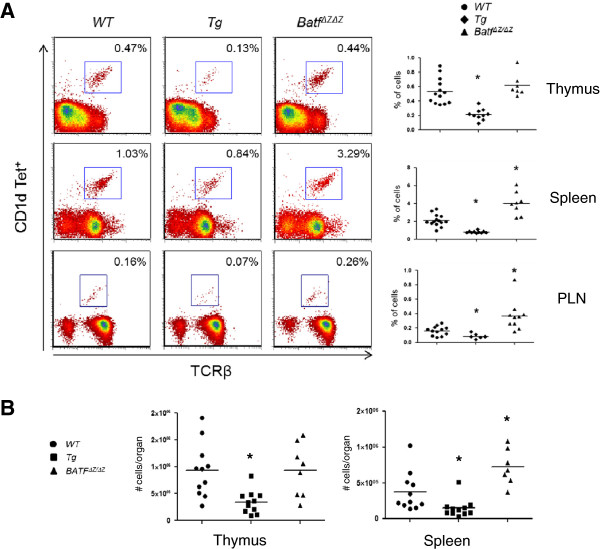
**BATF alters iNKT cells numbers *in vivo*. A** iNKT cells in single cell suspensions prepared from thymus, spleen and PLN of the indicated genotypes were detected by flow cytometry using glycolipid-loaded mouse CD1d tetramers. Cells gated are B220^-^ and iNKT cells are identified as TCRβ^+^ Tet^+^ (box). Representative flow plots are shown with % iNKT cells indicated. The mean and distribution of data obtained from at least 6 mice per genotype are presented on the right (n ≥ 6). *p values < 0.05. **B** Analysis of iNKT cells was performed as described in **A** and data expressed as total number of cells per indicated organ. The analysis of PLN was not performed. The mean and distribution of data obtained from the organs of at least 7 mice per genotype (n ≥ 7) are presented. *p values < 0.05.

Mice overexpressing BATF in thymic T cells (*p56*^*lck*^*HA-BATF*) display a reduced number of iNKT cells [6]. Interestingly, quantification of thymic iNKT cells in *Batf*^*ΔZ/ΔZ*^ mice revealed a number equivalent to WT mice [2]. As the *CD2-HA-BATF* transgene directs BATF overexpression to all T cells (thymic and peripheral) [7], iNKT cells were re-examined and compared to numbers in WT and *Batf*^*ΔZ/ΔZ*^ mice. The percentage of T cells positive for interaction with glycolipid-loaded CD1d tetramers was assessed by flow cytometry using single cell suspensions from thymus, spleen and PLN. Whether calculated on the basis of percentage (Figure 2A) or absolute number (Figure 2B), the results confirm that iNKT cells are under-represented in the thymus of Tg mice and extend that observation by showing fewer iNKT cells in the spleen and PLN as well. These data confirm that BATF deficiency does not impact the iNKT cell population in the thymus, yet do reveal, for the first time, that *Batf*^*ΔZ/ΔZ*^ mice possess a statistically significant increase in the number of peripheral iNKT cells.

**Figure 3:**
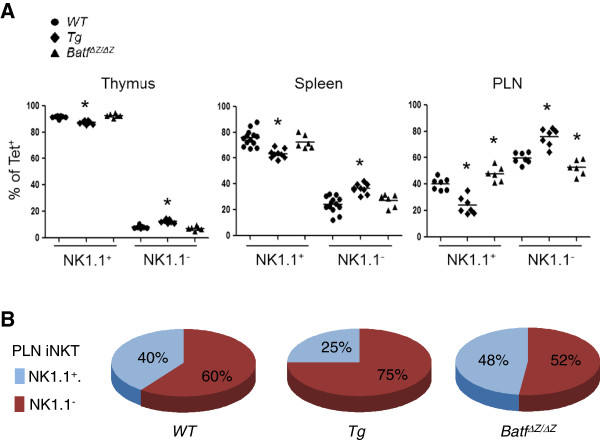
**BATF levels correlate with of NK1.1**^**- **^**iNKT cells. A** NK1.1^+^ and NK1.1^-^ iNKT cells were detected in cell suspensions from the indicated tissues using anti-NK1.1. Shown are the mean and distribution of the percent Tet^+^ NK1.1^+^ versus Tet^+^ NK1.1^-^ cells for at least 6 mice per genotype (n ≥ 6). *p values < 0.05. **B** Mean data from PLN in (**A**) were rounded to the nearest percent and presented in pie chart format to highlight the skewing of iNKT cell populations associated with different levels of BATF expression *in vivo.*

In previous studies, we reported that the residual iNKT cells in *p56*^*lck*^*HA-BATF* mice were disproportionately CD44^+^ NK1.1^-^ and concluded that BATF negatively influences iNKT cell expansion and maturation [9]. However, recent studies have identified an important sub-population of NK1.1^-^ iNKT cells that are fully mature and function in the periphery to regulate autoimmunity, inflammation and the host response to infection [13]. To profile NK1.1^-^ versus NK1.1^+^ iNKT cells in Tg, WT and *Batf*^*ΔZ/ΔZ*^ mice, the flow analysis was repeated to include an evaluation of NK1.1. As shown in Figure 3A, Tg mice with a reduced number of iNKT cells show a higher percentage of NK1.1^-^ cells in all tissues. Conversely, *Batf*^*ΔZ/ΔZ*^ mice show no bias in the distribution of NK1.1^-^ versus NK1.1^+^ iNKT cells in thymus and spleen, but a bias toward NK1.1^+^ in PLN. A graphic representation of the findings from PLN highlight the influence of BATF levels on the distribution of NK1.1^+^ versus NK1.1^-^ iNKT cells in the periphery (Figure 3B).

### Batf regulates the production of IL-17 by iNKT cells

**Figure 4:**
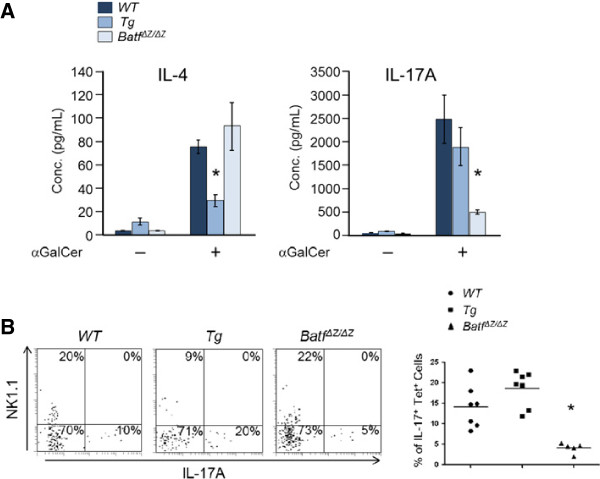
**BATF regulates IL-17 production by primary iNKT cells. A** Glycolipid-stimulated splenocytes of the indicated genotypes were analyzed by ELISA for secreted levels of IL-4 and IL-17A after 72 h. Graphed are data averaged from 6 mice per group (n = 6). Bars indicate standard error. *p values < 0.05. **B** iNKT cells were enriched from pooled PLN of the indicated genotypes, stimulated for 4 h, and NK1.1^-^ IL-17A^+^ cells quantified by flow cytometry. Representative plots gated on tetramer^+^ cells are shown on the left with the % of cells in each quadrant indicated. The mean and distribution of data from at least 5 mice per genotype are presented on the right (n ≥ 5). *p values < 0.05.

Peripheral NK1.1^-^ iNKT cells respond rapidly following stimulation and produce large quantities of IL-17 [10-12]. These cells are considered a distinct lineage and are designated as iNKT-17 cells [13]. The fact that iNKT cells in *CD2-HA-BATF* mice are predominantly NK1.1^-^, together with the observation that Tg animals display increased IL-17 expression and CD4^+^Th17 cell numbers, suggest that BATF may regulate the differentiation of iNKT-17 cells. To examine cytokine expression by iNKT cells, splenocytes from Tg, WT and *Batf*^*ΔZ/ΔZ*^ mice were stimulated *in vitro* with glycolipid antigen (αGalCer) and ELISA were performed (Figure 4A). As expected, IL-4 is secreted efficiently by iNKT cells in WT and in *Batf*^*ΔZ/ΔZ *^cultures, but accumulates to a lower level in Tg cultures where there are significantly fewer iNKT cells (Figure 2). For IL-17, the level of IL-17A secreted by Tg cultures was statistically indistinguishable from WT (Figure 4A), indicating that NK1.1^-^ cells overexpressing BATF readily produce this cytokine. On the other hand, IL-17A production by *Batf*^*ΔZ/ΔZ*^ cells, where NK1.1^-^ and NK1.1^+^ iNKT cells are present in roughly equal amounts, is dramatically less than IL-17A production by WT cells (Figure 4A). These data indicate that not only does BATF influence iNKT cell numbers, it is a critical determinant of IL-17 expression by iNKT cells. Flow cytometric analysis using intracellular staining to detect IL-17A in purified, stimulated iNKT cells from the PNL of Tg, WT and *Batf*^*ΔZ/ΔZ*^ mice provided additional evidence in support of this conclusion (Figure 4B).

### DN32.D3 cells model IL-17 production by iNKT cells

IRF4/BATF protein complexes bind to DNA within the mouse *Il17a/f* locus and are essential for the transactivation of the *Il17a/f* gene in Th17 cells [8]. However, the mechanism by which BATF functions to regulate gene expression in different cellular contexts, including iNKT-17 cells where IRF4 does not play a transcriptional role [18], continues to be investigated. Additionally, BATF and its interaction partner proteins such as JUNB and the IRF4 and 8 proteins [8,17], are expressed independently of IL-17 status in other T cell lineages [1,9] and BATF-containing protein complexes bind directly to genes where the transcriptional outcome is the inhibition of gene expression [1,4,19,20]. These facts complicate proposing a unified model to explain how BATF regulates its target genes. Therefore, to address the role of BATF in regulating *Il17a* and other target genes in iNKT cells, we sought to identify an *in vitro* system that could be used for this purpose.

**Figure 5:**
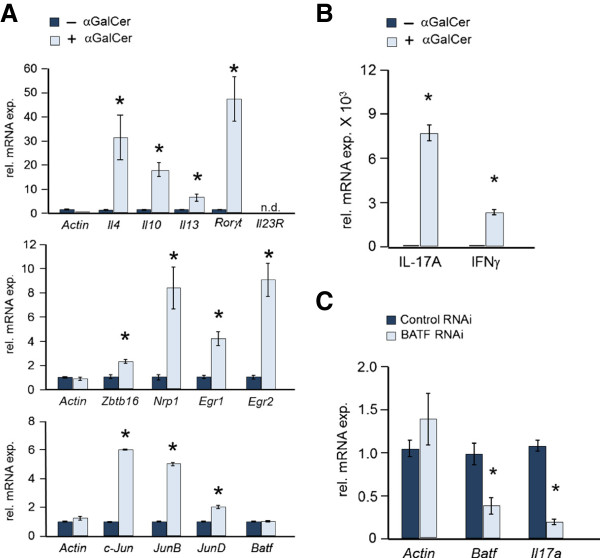
**DN32.D3 hybridoma cells display features of iNKT-17 cells. A** DN32.D3 cells were stimulated with plate-bound mouse CD1d dimers either loaded with aGalCer (+) or not (−) and after 24 h, RNA was prepared and analyzed by qRT-PCR for the indicated transcripts. Shown are the averages of 4 samples (n = 4). Bars indicate standard error. *p values < 0.05. **B** DN32.D3 cultures stimulated as in (**A**) for 24 h and RNA analyzed for *Il17a* and *Ifnγ* transcripts. The mean of 4 samples is presented (n = 4). Bars indicate standard error. *p < 0.05. **C** DN32.D3 cells were treated with siRNA for 24 h, washed and stimulated as in (**B**) for 2 h. RNA was isolated and analyzed for the indicated transcripts. Data shown are averaged from 5 samples (n = 5). Bars indicate standard error. *p values < 0.05.

DN32.D3 cells are a murine CD4^-^ CD8^-^ iNKT cell hybridoma [21] that have been used to investigate the ligand specificity of the iNKT TCR [22]. In response to stimulation, DN32.D3 cells express IL-2 [23] and up-regulate genes (*Il4, Il10, Il13*) encoding cytokines that define classic, iNKT cells (Figure 5A, top panel). Stimulation with αGalCer also leads to the induction of several genes essential for iNKT cell development (*Zbtb16 (Plzf), Nrp1, Egr1, Egr 2*) [13,24] (Figure 5A, middle panel). Interestingly, stimulation with glycolipid does not change the level of endogenous *Batf* gene expression, but instead increases expression of the genes encoding the basic leucine zipper dimerization partners of BATF (*c-Jun*, *JunB* and *JunD*) (Figure 5A, bottom panel). This up-regulation of JUN family members explains the increase in AP-1 DNA binding observed previously in αGalCer stimulated DN32.D3 cells [9]. Stimulated DN32.D3 cells express *Rorγt* mRNA (Figure 5A) and a very high level of *Il17a* mRNA (Figure 5B) which are shared features of the Th17 and iNKT-17 lineages. However, DN32.D3 cannot be classified as true iNKT-17 cells, as they are NK1.1^+^[21], express *Ifnγ* mRNA when stimulated and do not express *Il23R* (Figure 5A and C). Nevertheless, it is worth testing the role of BATF as a regulator of IL-17A expression in these cells.

Toward that goal, DN32.D3 cells were treated with *Batf* siRNA or control siRNA, stimulated with αGalCer loaded dimers and after 2 hr, RNA was analyzed by qRT-PCR. *Batf* siRNA resulted in a 50% reduction in *Batf* mRNA expression as well as a corresponding reduction in the level of *Il17a* mRNA (Figure 5C). These data support a critical role for BATF in *Il17a* gene regulation while demonstrating that signaling through the iNKT cell TCR expressed by DN32.D3 cells triggers cooperating molecular events that are required for efficient IL-17 induction. We conclude that the DN32.D3 iNKT cell line is a convenient *in vitro* model system in which the details of these molecular events can be investigated further.

## Conclusions

Our studies have identified BATF as a common regulator of lineage decisions in the murine immune system that involve the expression of IL-17. BATF joins RORγt in the transcription factor network that promotes the differentiation of IL-17 expressing cells downstream of pathways triggered by TGFβ and IL-6 in T cells [25] and by a pathway dependent on TGFβ, but not IL-6, in iNKT cells [11,26]. These studies have characterized the DN32.D3 iNKT cell line as a model in which the regulation of *Il17a* gene and protein expression by this lineage can be investigated further. As the molecular details controlling IL-17 production *in vivo* continue to emerge, new approaches to control autoimmunity, inflammation and infectious disease will become a reality.

## Methods

### Mice

*Batf*^*ΔZ/ΔZ*^ and *CD2-HA-BATF* mice expressing human, HA-tagged BATF were described previously [2,7] and were maintained by breeding to C57Bl/6 mice (Harlan, Indianapolis, IN). Experiments were performed using sex-matched littermates between 6 and 12 wk of age. Mice were housed in a specific, pathogen-free facility. All protocols were approved by the Purdue University Animal Care and Use Committee.

### Antibodies and reagents

All antibodies, cytokines and the mouse CD1d dimers were obtained from BD Biosciences unless otherwise specified. αGalCer was obtained from Axxora (Farmingdale, NY). PE-labeled, CD1d tetramers, empty or loaded with PBS-57, were obtained from the NIH tetramer core facility.

### Cell culture and the analysis of gene expression

CD4^+^ T cells were isolated using MACS (Miltenyi Biotec, Auburn, CA). Cells were stimulated with anti-CD3 and anti-CD28 Ab for 48 h as described [1]. For *in vitro* differentiation assays, naïve CD4^+^ T cells were isolated using MACS and cultured for 5 d under Th1 conditions [2] or under the following conditions for Th17: 5 μg/ml anti-CD3ε, 2 μg/ml anti-CD28, 5 ng/ml rh TGFβ, 100 ng/ml rm IL-6 and 10 μg/ml anti-IL-4 and 10 μg/ml anti-IFNγ (BioXCell, West Lebanon, NH) neutralizing antibodies. Cells were re-stimulated for 6 hr with 2 μg/ml anti-CD3ε prior to analysis. DN32.D3 cells were cultured as described [9] and stimulated for 24 h with plate-bound CD1d dimers +/− glycolipid prior to analysis.

RNA was isolated using Trizol and analyzed by qRT-PCR using SYBR green (Roche Diagnostics, Indianapolis, IN) and an ABI 7300 real-time PCR system. Data were normalized to *Hprt* expression and relative mRNA levels calculated using ΔΔCt values. The primers for *Hprt, Actin, Batf, Il4, Il23R, Il17a* and *Il21* have been described [2]. *Il10* primers were from Qiagen (Gaithersburg, MD) and additional primers (5’-3’) were

*Rorγt*: For TGTCCTGGGCTACCCTACTG, Rev GTGCAGGAGTAGGCCACATT;

*Ifnγ*: For GGATGCATTCATGAGTATTGC, Rev CCTTTTCCGCTTCCTGAGG;

*cJun*: For CAGTCCAGCAATGGGCACATCA, Rev GGAAGCGTGTTCTGGCTATGCA;

*JunB*: For GACCTGCACAAGATGAACCACG, Rev ACTGCTGAGGTTGGTGTAGACG;

*JunD*: For ACCTGCACAAGCAAAGCCAGCT, Rev CGAAACTGCTCAGGTTGGCGTA;

*Zbtb16*: For CCCAGTTCTCAAAGGAGGATG, Rev TTCCCACACAGCAGACAGAAG;

*Nrp1*: For GCCTGCTTTCTTCTCTTGGTTTCA, Rev GCTCTGGGCACTGGGCTACA;

*Erg1*: For GAGGAGATGATGCTGCTGAG, Rev TGCTGCTGCTGCTATTACC;

*Erg2*: For CCTCCACTCACGCCACTCTC, Rev CACCACCTCCACTTGCTCCTG;

*Il13*: For GCTTATTGAGGAGCTGAGCAACA, Rev GGCCAGGTCCACACTCCATA

For BATF knock-down, DN32.D3 cells were cultured for 24 h in siRNA delivery media containing 1 μM BATF targeting (Accell siRNA smart pool) or non-targeting siRNA (Accell non-targeting siRNA 1) (Dharmacon/Thermo Fisher Scientific, Waltham, MA). Cells were washed and stimulated for 2 h prior to analysis as described above.

### ELISA

Splenocytes at a density of 5×10^6^ cells/ml were cultured with 100 ng/ml αGalCer for 72 h. Supernatants were analyzed for secreted IL-4 (BD) or IL-17A (eBiosciences, San Diego, CA) using manufacturers’ protocols.

### Flow cytometry

Axillary, brachial, inguinal, and popliteal lymph nodes were pooled to generate PLN cultures. iNKT cells were enriched from PLN using CD8 (Ly-2) and CD19 microbeads (Miltenyi) as described [11]. Cells were surface stained after blocking with FcBlock (BD Biosciences, Franklin Lakes, NJ) using APC-anti- TCRβ (eBioscience), FITC-anti-B220, PE-Cy5-anti-B220, PBS-57-PE-CD1d tetramers and PE-Cy7-anti-NK1.1. Intracellular staining was performed as described [9] using FITC-anti-IL17A (eBioscience) after stimulation for 4 h with 5 ng/ml PMA and 500 ng/ml ionomycin in the presence of Golgi-plug (BD). Data were collected on a Beckman Coulter FC500 and analyzed using FCS Express V3 software (DeNovo, Los Angeles, CA).

## Abbreviations

AP-1: Activator Protein-1; αGalCer: α-Galactosylceramide; PLN: Peripheral Lymph Node; qRT-PCR: quantitative Reverse Transcriptase Polymerase Chain Reaction

## Competing interests

The authors declare no conflict of interest.

## Authors’ contributions

KLJ-W and EJT designed the experiments and wrote the manuscript. KLJ-W performed all experiments. KLJ-W and SP analyzed the data and prepared the figures. All authors read and approved the final manuscript.
